# From dopamine to muscarine: xanomeline-trospium (KarXT) as a novel direction in the psychopharmacotherapy of schizophrenia

**DOI:** 10.3389/fphar.2026.1774437

**Published:** 2026-02-11

**Authors:** Kacper Żełabowski, Wojciech Pichowicz, Julia Dembowska, Maciej Szwajkowski, Daria Mykhailova, Grzegorz Wurm, Kamil Biedka, Katarzyna Błaszczyk, Patryk Piotrowski

**Affiliations:** 1 Department of Forensic Medicine, Scientific Society for Psychopharmacology, Wrocław Medical University, Wrocław, Poland; 2 Department of Biochemistry and Immunochemistry, Wroclaw Medical University, Wrocław, Poland; 3 Department of Physiology and Pathophysiology, Wrocław Medical University, Wrocław, Poland; 4 Department of Psychiatry, Wrocław Medical University, Wrocław, Poland

**Keywords:** antipsychotic drugs, cholinergic modulation, KarXT, psychopharmacotherapy, schizophrenia, xanomeline-trospium

## Abstract

Schizophrenia is a severe chronic mental disease with considerable prevalence and social load. Its treatment relies on both typical and atypical antipsychotics, whose primary mechanism of action involves dopamine D_2_ receptor blockade, with atypical agents additionally modulating serotonergic pathways, which partially improves tolerability but does not adequately address cognitive symptoms. Xanomeline–trospium (KarXT) is the first FDA-approved drug for schizophrenia treatment via cholinergic receptor modulation. The antipsychotic feature of xanomeline lies in its M_1_ and M_4_ muscarinic receptor agonism in the central nervous system. Trospium does not cross the blood–brain barrier and acts as a peripheral muscarinic antagonist, thereby reducing the side effects of xanomeline, which also exerts peripheral activity. In Phase II and III clinical trials conducted between 2016 and 2023 (EMERGENT-1 to EMERGENT-5), KarXT demonstrated statistically significant reductions in psychotic symptoms, measured by the Positive and Negative Syndrome Scale compared with placebo in short-term randomized studies, with moderate effect sizes, and sustained symptom improvement and stable tolerability over 52 weeks in long-term extension trials. They also reported a favorable tolerability profile, with frequent mild gastrointestinal side effects. An important finding is that KarXT does not affect dopaminergic transmission and therefore does not cause the expected side effects typically associated with D_2_ receptor blockers, including extrapyramidal reactions and hyperprolactinemia. This review presents the pharmacological rationale behind KarXT and the current clinical evidence for its efficacy, safety, and proposes a breakthrough in the schizophrenia therapy.

## 1 Introduction

Schizophrenia remains one of the most common psychiatric disorders. The World Health Organization (WHO) approximates that 24 million individuals worldwide are afflicted (Meyer et al., 2025), with a global point prevalence of 1 in 300 for the general population and 1 in 222 for adults. The disease most often begins in late adolescence and young adulthood, with men falling ill earlier than women (WHO, 2026). Over the past 2 decades, the prevalence and incidence of schizophrenia have risen by 37% and 65%, respectively (Vasiliu et al., 2024). Schizophrenia consistently ranks among ten highest contributors to disability globally (JMCP, 2026), indicating the imperative need for novel therapeutic strategies (Vasiliu et al., 2024).

The standard treatment of schizophrenia is based on dopaminergic antipsychotic medication, commonly associated with many side effects. These include metabolic syndrome, hyperprolactinemia, QT interval prolongation, extrapyramidal symptoms, and malignant neuroleptic syndrome (JMCP, 2026; Ramey and Almodóvar, 2025). As a result, antipsychotic treatment may be limited by those effects, often contributing to poor adherence. Non-adherence, in turn, exacerbates the clinical symptoms of schizophrenia and further reduces the overall effectiveness of therapy (Menegaz d et al., 2025).

Additionally, dopaminergic antipsychotics mainly suppress the positive symptoms of schizophrenia, leaving patients burdened by negative symptoms and cognitive impairments (Meyer et al., 2025; Jamanetwork, 2025). Studies report that more than 75 percent of patients under antipsychotics experience at least one side effect; women and polypharmacy users are at particular risk (Iversen et al., 2018).

D_2_ receptor overactivation was observed in the associative striatum of untreated patients with schizophrenia, which may be involved in the occurrence of schizophrenia symptoms (Saint-Georges et al., 2025). Also, recent observations of transgenic organisms showed that overexpressing D_2_ receptors in the striatum is relevant to cognitive impairment (Kegeles et al., 2010). Dopamine hypoactivity in the prefrontal cortex, caudate, and alterations of D_3_ receptor activity are probably responsible for the negative and cognitive symptoms of schizophrenia. The cognitive deficits are also linked to reduced dopamine transmission in the neocortex and other cortical structures (Foster et al., 2021; Collo et al., 2020). Saint-Georges et al. review findings suggest that the cholinergic system is implicated in the development of psychosis by controlling the dopamine release in the striatum (Saint-Georges et al., 2025). Especially, the cholinergic M_1_ and M_4_ receptors show a strong relationship with the regulation of dopamine secretion (Dean et al., 2023). Neuroimaging and post-mortem studies demonstrate a link to dysregulated acetylcholine function in the pathomechanism of schizophrenia (Saint-Georges et al., 2025).

Acetylcholine is a key modulator in cortical and subcortical neurotransmission through muscarinic acetylcholine receptors. Of the five muscarinic receptor subtypes (M_1_-M_5_), M_1_ and M_4_ receptors especially are relevant to the pathophysiology of schizophrenia, as they are involved in cognitive processing and dopaminergic regulation. M_1_ receptors are predominantly expressed in cortical and hippocampal pyramidal neurons coupled with G_q_ proteins, leading to phospholipase C activation and intracellular calcium signaling. M_4_ receptors are mostly found in striatal regions and on presynaptic dopaminergic terminals, where their activation inhibits dopamine release via G_i/o_ protein–mediated pathways. This complementary modulation of cortical and subcortical circuits provides a mechanistic framework for targeting muscarinic receptors as an alternative to direct dopamine receptor antagonism in schizophrenia (Yohn et al., 2022).

Among patients on standard antipsychotic drug treatment, about 30% develop resistance, of which less than half would respond to clozapine, a drug for treatment-resistant schizophrenia (Vasiliu et al., 2024; Smith et al., 2025). Using clozapine is associated with serious side effects, including agranulocytosis, myocarditis, seizures, and typical symptoms such as drowsiness, weight gain, excessive salivation, and constipation. Due to the risk of serious complications, clozapine was withdrawn from the market in 1976. In 1990-1991, after studies confirmed its effectiveness, it was reapproved for use (Wright et al., 2024).

Xanomeline-trospium (KarXT) is a combination of xanomeline, an M_1_ and M_4_ receptor agonist, and trospium chloride, a peripheral cholinergic receptor antagonist with no ability to cross the blood-brain barrier (Vasiliu et al., 2024). Muscarinic M_1_ and M_4_ receptors are instrumental in controlling glutamatergic and dopaminergic pathways. This neuromodulatory strategy achieves antipsychotic efficacy, limiting adverse effects typically associated with D_2_ receptor antagonism, as KarXT does not directly interfere with dopamine pathways (Hasan and Abid, 2024).

Trospium, by its antagonistic activity, reduces the incidence of xanomeline-induced peripheral cholinergic adverse effects (Azargoonjahromi, 2024). In various studies, KarXT effectively reduced psychotic symptoms of schizophrenia and was generally well tolerated (Jamanetwork, 2025; Correll et al., 2023; Kaul et al., 2025; Yeung et al., 2025). By modulating cholinergic system activity, it also influences GABAergic transmission, which is involved in the regulation of cognitive function, psychosis, and addiction (Vasiliu et al., 2024). This approach represents a new treatment paradigm focused on restoring the physiological balance of neurotransmitters.

Efficacy was assesed using changes in Positive and Negative Syndrome Scale (PANSS), a scale designed to asses symptoms of schizophrenia. Reductions in PANSS scores are generally associated with effective symptom control, whereas minimal or absent changes may indicate limited therapeutic efficacy or treatment resistance. A 20%–30% reduction in PANSS total score is generally considered to represent a clinically meaningful improvement, while a ≥30% reduction indicates a clear and clinically noticeable response (Si et al., 2021; Leucht et al., 2019).

The second scale which was used is Clinical Global Impression–Severity scale (CGI-S). It assesses the clinical severity of schizophrenia at a given time point. It provides global clinical evaluation that complements a specified scale, such as PANSS. It is a 7-point scale, thus the change of even one point is clinically significant (Si et al., 2021).

## 2 Methodology

This narrative review aims to provide a comprehensive overview of the current knowledge about KarXT in schizophrenia treatment. To identify the most relevant clinical evidence regarding the efficacy of KarXT, an extensive literature search was performed in various academic databases: PubMed, Scopus, Embase, ClinicalTrials.gov, and Web of Science.

The inclusion criteria involve clinical studies performed on adult patients diagnosed with schizophrenia (according to ICD-10, DSM-5, or DSM-IV); studies performed in the exacerbation of schizophrenia and focusing on KarXT in the context of schizophrenia psychopharmacotherapy. Due to the novelty of the drug KarXT, we did not impose a publication date regime, and we also included older papers on xanomeline and trospium as drugs used in treatment relative to each other independently.

The exclusion criteria include preclinical or animal-only studies, studies focused on unrelated indications, and use of KarXT in a non-standard formulation.

We used the following keywords: xanomeline-trospium, KarXT, xanomeline, trospium, Cobenfy, muscarinic receptors, cholinergic modulation, schizophrenia, psychosis, PANSS score.

The most important information was obtained and presented in a narrative approach, with particular emphasis on pharmacokinetic aspects, receptor selectivity, and clinical trial results. In addition, the quality and validity of scientific research were taken into account in order to present a reliable and up-to-date state of knowledge on the topic presented.

All works meeting our criteria turned out to be in English only.

## 3 KarXT as an example of cholinergic modulation in the treatment of schizophrenia

KarXT represents a novel therapeutic strategy in schizophrenia by selectively targeting central muscarinic acetylcholine receptors, thereby offering a non-dopaminergic approach to symptom control. Unlike conventional antipsychotics, its mechanism of action is grounded in cholinergic modulation, addressing long-standing pathophysiological hypotheses implicating muscarinic signaling in psychosis (Dean and Scarr, 2020).

Accordingly, this section outlines KarXT as a case example of cholinergic modulation in schizophrenia, progressing from its neurobiological and pharmacokinetic rationale (Section 3.1), through evidence on clinical efficacy, safety, and tolerability (Section 3.2), to recent formulation advances designed to optimize its clinical usability (Section 3.3).

Xanomeline is a drug originally developed in the 1990s as a new substance with potential in the treatment of Alzheimer’s disease. At the clinical trial stage, the drug has shown beneficial effects on cognitive function and has reduced psychotic symptoms in dementia patients (Bodick et al., 1997; Fisher, 2025; Avery et al., 1997). However, due to persistent side effects associated with activation of peripheral Muscarinic Acetylcholine Receptors (mAChRs) - mainly from the gastrointestinal and autonomic systems (i.e., diarrhea, sweating, nausea) - studies on its clinical use were discontinued (Shekhar et al., 2008).

The most important clinical challenge was to reduce the peripheral side effects of muscarinic receptor agonism without compromising the beneficial central effects of xanomeline. In this context, trospium - previously used in the management of overactive bladder (OAB) - has been applied (Staskin et al., 2010). The resulting fixed-dose drug combination, KarXT, was officially registered by the FDA on 26 September 2024, for the treatment of schizophrenia in adults (Commissioner of the FDA, 2024). Karuna Therapeutics is responsible for the pharmaceutical combination, selling the product under the name Cobenfy™; the prefix “Kar” in KarXT reflects the company’s name, while “XT” denotes its components (FDA, 2025). Available meta-analyses indicate that KarXT has a promising therapeutic profile, including high clinical efficacy, favorable safety of use, and good tolerability in patients with schizophrenia (Fabiano et al., 2025; Mohammed et al., 2025; Guo et al., 2025). Specifically, the meta-analysis by Menegaz de Almeida et al. identified premature discontinuation rates primarily driven by gastrointestinal adverse events, especially nausea and vomiting, despite overall favorable efficacy outcomes. These findings highlight tolerability as a clinically relevant limitation, rather than lack of antipsychotic effectiveness (Menegaz d et al., 2025). Similarly, Wright AC et al. emphasized that evidence regarding long-term durability of symptom control, adherence, and rare adverse events remains limited. The authors underscored the need for extended-duration studies to better characterize KarXT’s role in chronic schizophrenia management and to facilitate comparisons with established long-term antipsychotic therapies (Wright et al., 2024).

Xanomeline exhibits high affinity for muscarinic M1 and M4 receptors. Its functional relevance in schizophrenia is attributed to expression and involvement of these receptors in key brain regions implicated in psychosis and cognition, including the cerebral cortex, hippocampus, and nucleus accumbens (Thorn et al., 2019). Owing to its ability to cross the blood–brain barrier, xanomeline modulates central cholinergic signaling, which has been associated with improvements in cognitive performance and reductions in psychotic symptoms such as delusions and hallucinations in clinical studies (Shekhar et al., 2008; Guo et al., 2025; Harvey et al., 2025).

Trospium, due to its hydrophilic (polar) structure, does not penetrate the blood-brain barrier (Rovner, 2004) and acts peripherally, primarily protecting the intestines, bladder, and sweat glands. It prevents side effects of xanomeline outside the central nervous system, including diarrhea, nausea, and increased sweating (Breier et al., 2023).

### 3.1 Neurobiological mechanisms and pharmacokinetics of KarXT

Muscarinic M_4_ receptor agonism is involved in the modulation of dopaminergic transmission through several mechanisms (Moehle and Conn, 2019). First, M_4_ receptor activation inhibits cholinergic projections from the hindbrain to the midbrain, reducing acetylcholine concentrations in the ventral tegmental area (VTA). This reduction consequently diminishes dopaminergic transmission within the associative striatum. In addition, activation of M_4_ receptors located on the terminals of cortical neurons within the associative striatum modulates corticostriatal input, which in turn further reduces striatal dopamine release. M_4_ receptors are also expressed on D_1_-type medium spiny neurons. Through their projections to the striatum, these neurons can trigger the release of endocannabinoids, which, by binding to cannabinoid receptor type 2 (CB_2_ receptors) on dopamine terminals, inhibit dopamine release via the endocannabinoid-CB2 signaling pathway (Covey et al., 2017). Moreover, M_4_ receptor activity is also relevant beyond the striatal circuits. Its neocortex stimulation, including posterior associative regions, and hippocampus may contribute in an integrated manner to Cobenfy’s cortical network modulation and cognitive processing (Dean et al., 2023; Felder et al., 2018).

Clinically, it is notable that the sensorimotor striatum does not exhibit the same high sensitivity to M_4_ receptor-mediated modulation. Therefore, xanomeline does not have any profound impact on the presynaptic dopamine release in this region. The absence of this activity results in a favorable safety profile, minimizing the risk of adverse effects related to motor function, such as Parkinsonism or akathisia, typical of D_2_ dopamine receptor antagonists.

**FIGURE 1:**
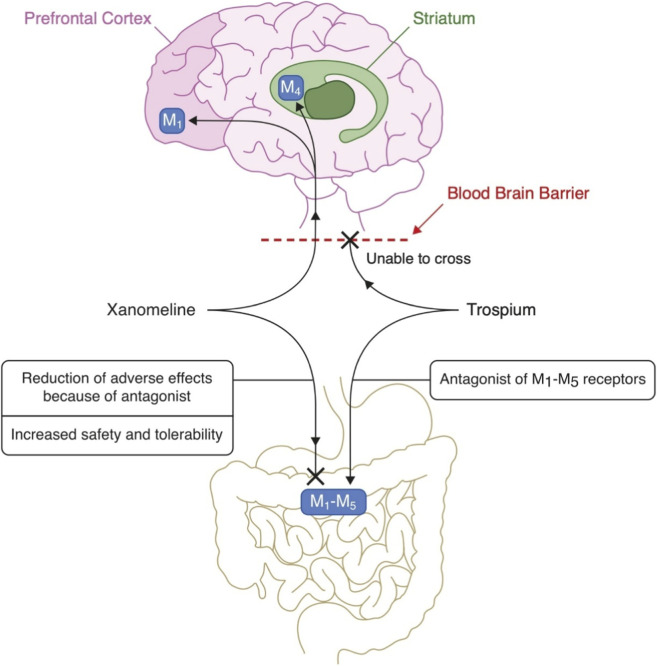
Proposed mechanism of KarXT: xanomeline crosses the blood-brain barrier and selectively activates central M_1_ and M_4_ receptors, while trospium acts peripherally, blocking M_1_, M_5_ receptors to prevent cholinergic side effects. KarXT, xanomeline–trospium; M_1_, muscarinic acetylcholine receptor subtype 1; M_4_, muscarinic acetylcholine receptor subtype 4; M_5_, muscarinic acetylcholine receptor subtype 5.

**FIGURE 2:**
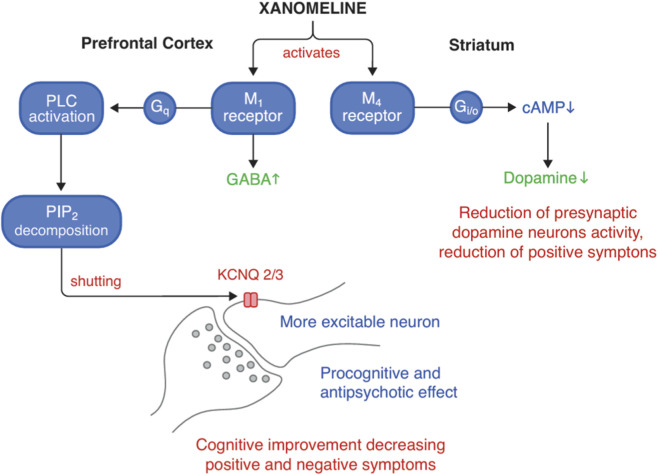
Proposed neurobiological mechanism of KarXT: M_1_ receptor activation in the prefrontal cortex enhances GABAergic transmission and neuronal excitability, contributing to cognitive improvement; M_4_ receptor activation in the striatum reduces presynaptic dopamine release, alleviating positive symptoms. cAMP, cyclic adenosine monophosphate; GABA, gamma-aminobutyric acid; G_i/o_–coupled receptors inhibit adenylyl cyclase; G_q_–coupled receptors activate PLC; KarXT, xanomeline–trospium; KCNQ 2/3, subunits 2 and 3 of potassium voltage-gated channel; M_1_, muscarinic acetylcholine receptor subtype 1; M_4_, muscarinic acetylcholine receptor subtype 4; PIP_2_, phosphatidylinositol 4,5-bisphosphate; PLC, phospholipase C.

In the prefrontal cortex, xanomeline acts as an M_1_ receptor agonist. The enhanced cholinergic activity then activates GABAergic interneurons, whose stimulation modulates the glutamatergic pathway descending to the ventral striatum. The result is an increase in dopamine release dependent on glutamate activity. The described mechanism may explain both the antipsychotic and potentially pro-cognitive effects of Xanomeline (Yohn et al., 2022; Paul et al., 2024; Li et al., 2008). The visualization of KarXT pharmacodynamics and action mechanism is presented in Figures 1, 2.

Xanomeline is rapidly metabolized and shows very low bioavailability (<1%) due to intensive hepatic first-pass metabolism (Mirza et al., 2003; Davie et al., 2013). Consequently, its plasma half-life is about 5 h, and most of the dose is excreted by the kidneys within 24 h of administration (Lobo et al., 2022). The maximum plasma concentration (C_max_) is 8.95 and 13.81 ng/mL (after doses of 100 and 150 mg, respectively), and peak plasma drug concentration is achieved in about 2.4–2.5 h (T_max_) (Mirza et al., 2003; Lucas et al., 1995).

Trospium is metabolized only to a small extent; bioavailability is approximately 10% and decreases with concomitant food intake (to about 26% of the surface area under plasma in the fasting state). The peak plasma concentration (C_max_) of about 4 ng/mL is reached after the fourth hour (T_max_) after administration of 20 mg of the immediate release preparation (Doroshyenko et al., 2005; Silver et al., 2010; Zhang et al., 2012). Trospium does not cross the blood-brain barrier due to its size, charge, and hydrophilic properties (Çetinel and Onal, 2013). The average elimination half-life is 10–20 h. The main metabolites are spiroalcohol and hydrolysis/oxidation products, neither of which is pharmacologically active. Most of the drug is excreted in the urine (Doroshyenko et al., 2005).

The bioavailability of xanomeline administered with trospium was similar in healthy volunteers compared to patients with schizophrenia. In addition, when administered with trospium, xanomeline reached 10% higher plasma concentrations compared to xanomeline alone (Singh, 2022). Analysis of the pharmacokinetic profile of trospium showed that it did not affect the pharmacokinetic profile of xanomeline. The addition of trospium to xanomeline reduced cholinergic side effects compared to xanomeline alone (Breier et al., 2023).

However, an important limitation is that trospium itself must be administered under fasting conditions: concomitant food intake reduces trospium bioavailability by 70%–80% (Cvijić and Langguth, 2014), leading to impaired absorption and decreased peripheral antagonistic effect. As a consequence, although xanomeline exposure is enhanced in the presence of trospium, inadequate trospium absorption in non-fasting states may diminish tolerability benefits by insufficiently mitigating peripheral cholinergic effects. This strict requirement to take KarXT on an empty stomach is a significant practical barrier for many patients and has been suggested as a possible contributor to higher discontinuation rates in real-world settings. Detailed pharmacokinetic parameters of both agents are presented in Table 1.

**TABLE 1:** Pharmacological characteristics of xanomeline and trospium: components of the KarXT combination in schizophrenia treatment.

Characteristic	Medical indication^5^	Route of administratio^2,5^	Bioavailability^1,2^	Protein binding^1,2^	C_max_^1,2^	T_max_^1,2^	Main metabolites^3,4^	Elimination^1,2^	Half-life (T½)^1,2^	Chemical structure
Xanomeline	Schizophrenia (CNS muscarinic M_1_/M_4_ agonist component of KarXT)	Oral (in combination with trospium)	Very low (<1%)	Approximately 95%	8.95 and 13.81 ng/mL (after doses of 100 and 150 mg, respectively)	∼2.4–2.5 h	Extensive hepatic metabolism; specific metabolites not detailed	Primarily renal; most drugs are excreted within 24 h	∼5 h	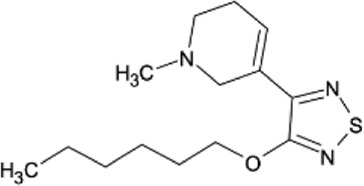
Trospium	Mitigation of peripheral cholinergic side effects in KarXT (M antagonist)	Oral	Modestly (10%)	Approximately 50%	4 ng/mL (after 20 mg dose)	∼4–5 h	Spiroalcohol and hydrolysis/oxidation products (non-active)	Primarily renal	∼10–20 h	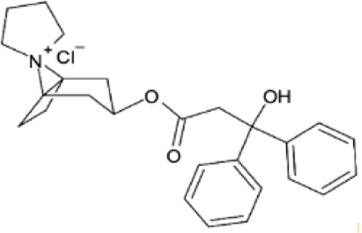

C_max_, maximum plasma concentration; CNS, central nervous system; *KarXT, xanomeline-trospium;* M_1_, muscarinic acetylcholine receptor subtype 1; M_4_, muscarinic acetylcholine receptor subtype 4; T_max_, time to maximum plasma concentration; T½, elimination half-life.

Primary references:

1. Early clinical pharmacokinetic studies of Xanomeline (Mirza et al., 2003; Lucas et al., 1995)
2. Trospium chloride - Prescribing Information (FDA) (FDA, 2025)
3. Xanomeline metabolism and biotransformation studies (Mirza et al., 2003; Lucas et al., 1995; DeLong et al., 1995)
4. Trospium chloride - regulatory metabolism data (FDA, 2025; Doroshyenko et al., 2005)
5. Clinical development and mechanism of action of KarXT (Meyer et al., 2025; Yohn et al., 2022; Commissioner of the FDA, 2024).

### 3.2 Clinical efficacy, safety, and tolerability

Approval of KarXT was preceded by Phase II clinical trials - EMERGENT-1 (n = 182) and Phase III clinical trials - EMERGENT-2 (n = 252), EMERGENT-3 (n = 256) conducted by biotechnology company Karuna Therapeutics. The EMERGENT-1, EMERGENT-2, and EMERGENT-3 studies were randomized, double-blind, placebo-controlled trials. They involved a total of 690 adult patients with a diagnosis of schizophrenia who were experiencing an increase in psychotic symptoms and required hospitalization (Leber et al., 2024; Psychiatrictimes, 2025).

Patients were randomized 1:1 to KarXT or placebo. The EMERGENT-1, EMERGENT-2 and EMERGENT-3 trials used the same treatment regimen. The study lasted 5 weeks and consisted of administering the drug twice a day, with doses increasing gradually. On days 1–2, patients took 50 mg of xanomeline and 20 mg of trospium, on days 3–7 - 100 mg of xanomeline and 20 mg of trospium, increasing the dose up to 125 mg xanomeline and 30 mg trospium on subsequent days; with a possible dose reduction in case of intolerance (Kaul et al., 2024a).

In the EMERGENT-1 study, the group of patients receiving KarXT demonstrated a mean reduction of 20.6 points, while the placebo group exhibited a reduction of 12.2 points. The observed difference between the groups was 8.4 points (p < 0.001), which corresponds to an effect size of 0.60. This value represents the mean effect (Kaul et al., 2025).

Additional *post hoc* analyses of the EMERGENT-1 trial also showed significant improvements in cognitive function in patients with cognitive deficits treated with KarXT compared to placebo. Effects were observed in all five symptom domains assessed by the PANSS scale, including positive symptoms, negative symptoms, thought disorganization, anxiety/depression, and uncontrolled hostility (Yeung et al., 2025).

Results from the EMERGENT-2 trial showed that KarXT statistically and clinically significantly reduced the PANSS total score by 9.6 points compared to placebo, with a decrease from 21.2 points to 11.6 (p < 0.0001). The results of the two independent studies (EMERGENT-1 and EMERGENT-2) are consistent, strengthening their reliability. Results from EMERGENT-3 also showed that KarXT statistically and clinically significantly reduced the PANSS total score by 8.4 points compared to placebo after 5 weeks of the study. The reduction from 20.6 to 12.2 (p < 0.0001, Cohen’s d coefficient was 0.60) (Kaul et al., 2024b).

The use of KarXT in the treatment of schizophrenia led to a significant improvement in the PANSS scale. The mean difference in change based on the three studies was −9.9 points compared to placebo (p < 0.0001), corresponding to an effect size of 0.65, which falls within the moderate/major effect range (Kaul et al., 2025).

In addition to analyzing the effect of treatment on positive and negative symptoms of the disease, these studies provide data on the side effect profile and tolerability of therapy, comparing results in the KarXT-treated group with the placebo-treated group (Kaul et al., 2024a; Kaul et al., 2024b; Brannan et al., 2021). Across all of the EMERGENT-1-3 studies, most reported adverse reactions include gastrointestinal symptoms: nausea, vomiting, dyspepsia, constipation; usually of mild to moderate severity and transient in nature. Transient elevations in blood pressure and heart rate have also been observed in a small percentage of patients and require further observation in long-term safety studies. Most reported adverse events in Phase II and III studies were summarized in Table 2.

**TABLE 2:** Most common adverse events of KarXT therapy.

Medication(s)	Type of study	Duration	Participants (KarXT vs. placebo)	Treatment effects (KarXT vs. placebo)	References
KarXT vs. placebo	Phase II RCT (EMERGENT-1)	5 weeks	89 vs. 90	Most common adverse events • nausea 15 (17%) vs. 4 (4%) • vomiting 8 (9%) vs. 4 (4%) • constipation 16 (18%) vs. 3 (3%) • dry mouth 8 (9%) vs. 1 (1%) Most adverse events were mild–moderate; no severe cholinergic/anticholinergic events	Correll et al. (2022)
KarXT vs. placebo	Phase III RCT (EMERGENT-2)	5 weeks	126 vs. 126	Most common adverse events • constipation 27 (21%) vs. 13 (10%) • dyspepsia 24 (19%) vs. 10 (8%) • headache 17 (14%) vs. 15 (12%) • nausea 24 (19%) vs. 7 (6%); vomiting 18 (14%) vs. 1 (1%) • hypertension 12 (10%) vs. 1 (1%) • dizziness 11 (9%) vs. 4 (3%) EPS/akathisia/weight gain rates similar. Discontinuations: 7% vs. 6%	Kaul et al. (2024a)
KarXT vs. placebo	Phase III RCT (EMERGENT-3)	5 weeks	125 vs. 131	Most common adverse events • nausea 24 (19%) vs. 2 (2%) • dyspepsia 20 (16%) vs. 2 (2%) • vomiting 20 (16%) vs. 1 (1%) • constipation 16 (13%) vs. 5 (4%) EPS, weight gain, and somnolence rates similar. Discontinuations due to TEAEs: 6.4% vs. 5.5%	Kaul et al. (2024b)

EMERGENT, program of clinical trials evaluating KarXT, in schizophrenia; EMERGENT-1, EMERGENT-2, EMERGENT-3, individual trials within the EMERGENT, program; EPS, extrapyramidal symptoms; KarXT, xanomeline-trospium; RCT, randomised controlled trial; TEAEs, treatment-emergent adverse events.

The EMERGENT-1, EMERGENT-2 and EMERGENT-3 studies were short-term studies, so they were expanded to include EMERGENT-4 (n = 126) and EMERGENT-5 (n = 125). These are Phase III, multicenter, open-label, 52-week studies evaluating the long-term safety, tolerability, and efficacy of therapy. The therapeutic doses administered to patients were analogous to those used in the EMERGENT-1, EMERGENT-2, and EMERGENT-3 studies (n = 89, n = 126, and n = 125) (Leber et al., 2024; Psychiatrictimes, 2025; Kaul et al., 2024a). The dosing regimen was as follows: 50 mg of xanomeline and 20 mg of trospium administered twice daily for the first 2 days; 100 mg of xanomeline and 20 mg of trospium administered twice daily on days 3–7; and from day 8 onward, flexible dosing with an optional increase to 125 mg of xanomeline and 30 mg of trospium twice daily, with the option to return to 100 mg of xanomeline and 20 mg of trospium based on tolerability (Psychiatrictimes, 2025).

Significant improvements in schizophrenia symptoms, as measured by the PANSS scale, were observed. The EMERGENT-4 study included 156 patients who had previously participated in EMERGENT-2 or EMERGENT-3. After 52 weeks of treatment with KarXT, 69% of participants achieved ≥30% improvement on the PANSS scale compared to baseline; there was a 22.1-point decrease from baseline on the PANSS (Psychiatrictimes, 2025).

The EMERGENT-5 study included 566 patients who had previously been stabilized on treatment with other antipsychotics. In the EMERGENT-5 study, after 52 weeks of treatment, more than 75% of participants achieved a reduction in PANSS scale scores of more than 30% compared to baseline. The average change was 33.3 PANSS scale points. In addition, the mean Clinical Global Impression-Severity (CGI-S) scale score improved by 1.7 points, representing a shift from the “significantly ill” to “moderately” or “mildly ill” category after 1 year of treatment (Psychiatrictimes, 2025). Both the EMERGENT-4 and EMERGENT-5 trials showed that KarXT is an effective and well-tolerated therapeutic option in the acute phase of schizophrenia.

The results of EMERGENT-4 and EMERGENT-5 did not reveal any new safety signals beyond the side effect profile identified in earlier Phase 1 and Phase 2 studies. In both studies, treatment with KarXT was well tolerated over 52 weeks. No increase in the frequency or severity of adverse effects was observed over the course of therapy, suggesting no cumulative toxicity and a stable tolerability profile for the drug in long-term use (Psychiatrictimes, 2025).

The most reported adverse effects in EMERGENT-1 included nausea, vomiting, constipation and dry mouth; constipation, dyspepsia, nausea and vomiting in EMERGENT-2; while EMERGENT-3 adverse effects were consistent with previous studies.

The data obtained, which agree with results from earlier stages of KarXT’s clinical development, indicate its favorable safety and tolerability profile in the chronic treatment of schizophrenia. In addition, these results support the clinical potential of KarXT as an alternative to conventional antipsychotics, particularly in terms of reducing side effects associated with dopamine receptor blockade. Key characteristics of the EMERGENT clinical trials are summarized in Table 3.

**TABLE 3:** Clinical trials evaluating the efficacy, safety, and tolerability of KarXT in schizophrenia.

Type of study	Duration	Number of all participants	Number of participants in the KarXT treatment group	Characteristics of treatment group	Treatment	Treatment effects	Adverse effects	Tolerance	References
Randomized, double-blind, placebo-controlled clinical trial (EMERGENT-1)	5 weeks	182	90	Adults with recent worsening of positive symptoms that warranted hospitalization	50 mg/20 mg twice daily, increasing to a maximum of 125 mg/30 mg twice daily	Study primarily focused on safety and tolerability	Nausea 16.9%, vomiting 9.0%, anticholinergic AEs constipation 16.9%, dry mouth 9.0%	KarXT demonstrated a favorable tolerability profile, adverse events were generally mild to moderate, and did not lead to study discontinuation, the addition of trospium mitigated peripheral cholinergic side effects without introducing significant central anticholinergic effects	Correll et al. (2022)
Phase 3 randomized, double-blind, placebo-controlled, clinical trial (EMERGENT-2)	5 weeks	252	126	Adults with schizophrenia experiencing acute psychosis, PANSS Total Score ≥80, CGI-S score ≥4	50 mg xanomeline and 20 mg trospium twice per day for the first 2 days, 100 mg xanomeline and 20 mg trospium twice per day for days 3–7. From day 8 flexible dosing with an optional increase to 125 mg xanomeline and 30 mg trospium twice per day with option to return to 100 mg xanomeline and 20 mg trospium based on tolerability	Statistically significant and clinically meaningful improvement in PANSS Total Score at week 5 compared to placebo, with improvements in secondary endpoints: PANSS positive subscale, PANSS negative subscale, PANSS Marder negative factor, Clinical Global Impression–Severity (CGI-S) score	≥5% and at least twice the rate of placebo: constipation, dyspepsia, nausea, vomiting, hypertension, dizziness, gastroesophageal reflux disease	Majority of TEAEs occurred within the first 2–3 weeks of treatment, and resolved before the end of the trial. Discontinuation rates due to TEAEs were similar between KarXT and placebo groups. Side effects were generally mild to moderate and consistent with the pharmacological action ofmuscarinic receptor modulation	Kaul et al. (2024a)
Phase 3 randomized, double-blind, placebo-controlled clinical trial (EMERGENT-3)	5 weeks	253	125	Adults with schizophrenia experiencing acute psychosis, PANSS Total Score ≥80, CGI-S score ≥4	50 mg xanomeline and 20 mg trospium twice per day for the first 2 days, 100 mg xanomeline and 20 mg trospium twice per day for days 3–7. From day 8 flexible dosing with an optional increase to 125 mg xanomeline and 30 mg trospium twice per day with option to return to 100 mg xanomeline and 20 mg trospium based on tolerability	Statistically significant and clinically meaningful improvement in PANSS Total Score at week 5 compared to placebo, with improvements also observed in secondary endpoints: PANSS positive subscale, PANSS negative subscale, Clinical Global Impression–Severity (CGI-S) score	Adverse effect profile substantially consistent with the previously reported EMERGENT-1 and EMERGENT-2 trials, most common treatment-emergent adverse events in the KarXT group (≥5% and at least twice the rate of placebo): constipation, dry mouth, nausea, dyspepsia	KarXT demonstrated a favorable tolerability profile with overall discontinuation rates similar between KarXT and placebo groups; side effects were generally mild to moderate and consistent with the pharmacological action of muscarinic receptor modulation	Kaul et al. (2024b)

AEs, adverse effects; CGI-S, Clinical Global Impression–Severity; EMERGENT, program of clinical trials evaluating KarXT, in schizophrenia; EMERGENT-1, EMERGENT-2, EMERGENT-3, individual trials within the EMERGENT, program; *KarXT, xanomeline-trospium;* PANSS, Positive and Negative Syndrome Scale; TEAEs, treatment-emergent adverse events.

Phase 3 ARISE study was a double blind, randomized, placebo-controlled trial conducted in adults with schizophrenia inadequately controlled on a stable atypical antipsychotic, where the efficacy and safety of Cobenfy was examined as an adjunctive drug. It did not meet the primary endpoint (LSMD in PANSS total −2.0 points vs. placebo addon at Week 6; p = 0.11), while safety was consistent with monotherapy trials. An exploratory *post hoc* subgroup analysis suggested nominal benefit when background treatment excluded risperidone, however, this was not multiplicity controlled (BMS, 2025).

Importantly, ARISE differed fundamentally from the EMERGENT program in both its clinical rationale and design. Whereas EMERGENT-1 through EMERGENT-3 investigated Cobenfy as monotherapy in patients undergoing acute psychotic exacerbations, ARISE targeted a distinct population and tested add-on efficacy in stable patients rather than confirming monotherapy results (Kaul et al., 2024a; Kaul et al., 2024b; Brannan et al., 2021; BMS, 2025).

Because of these conceptual and methodological differences, outcomes from ARISE cannot be directly compared with those of the EMERGENT trials to infer phase-to-phase continuity or mechanistic consistency. Consistent with this, ARISE did not meet its primary endpoint, while its safety profile was broadly consistent within the monotherapy program (Kaul et al., 2024a; Kaul et al., 2024b; Correll et al., 2022; BMS, 2025).

Overall, the negative primary outcome in ARISE is best understood as reflecting the difficulty of demonstrating short-term adjunctive benefit in stable patients with residual symptoms, rather than contradicting the robust efficacy observed in acute monotherapy settings (Leber et al., 2024; BMS, 2025).

### 3.3 TerXT–extended-release formulation (early-phase development)

Building on the established efficacy and safety profile of immediate-release KarXT, the TerXT extended-release formulation was developed to improve tolerability and dosing convenience while preserving therapeutic activity. It represents an early-stage, extended-release formulation of xanomeline–trospium, currently evaluated in Phase 1 studies, and is included here to illustrate formulation strategies rather than to infer clinical efficacy (TerXT, 2025).

KarXT is administered twice daily, what may pose a significant challenge for people struggling with schizophrenia as nonadherence is a known problem in this patient group. The gold standard for oral antipsychotics is once-daily dosing, as asserted by the TerXT developers at Terran Biosciences (TerXT, 2025).

TerXT is a novel fixed-dose combination formulation consisting of a xanomeline and trospium prodrugs - the structural design of this compound is intended to prolong the therapeutic effect compared to non-prodrug forms of xanomeline and trospium. Using the pro-drug approach, Terran Biosciences was able to develop new drug combinations for two distinct administration forms: once-daily oral administration (TerXT) and an extended-release form administered intramuscularly once every few weeks or months (TerXT LAI) (Kuntz and Clark, 2026).

Both solutions remain on track to enter Phase I clinical trials, which are expected to commence in the near future. Terran plans to conduct two “bridging” Phase I studies - one for each compound. The primary objectives are to confirm safety and pharmacokinetic properties and to demonstrate bioequivalence with the approved reference product. Successful completion of these studies would allow the use of the 505(b) (WHO, 2026) registration pathway for TerXT and TerXT LAI once the 5-year exclusivity period of BMS has expired (Beaney, 2024).

The 505(b) (WHO, 2026) pathway is intended for new chemical molecules that contain changes to drugs that have already been approved. This pro-drug approach has a documented history of its application on numerous occasions considering antipsychotic pro-drugs. In this particular instance, the NDA may include safety and efficacy data derived from studies not conducted by the applicant. Terran intends to utilize data from the Phase III KarXT BMS study (Beaney, 2024).

## 4 Discussion

Xanomeline-trospium represents a promising direction in the treatment of schizophrenia as the first FDA-approved based on cholinergic modulation. Thanks to its innovative mechanism of action, the drug reduces positive symptoms as well as brings significant improvements in cognitive deficits and negative symptoms, which have been a major therapeutic problem until now (Paul et al., 2024; Kaul et al., 2024a; Kaul et al., 2024b; Brannan et al., 2021; Weiden et al., 2022).

Short-term (5-week) studies reported statistically significant and clinically meaningful reductions in PANSS total score, with effect sizes of moderate to large (average d = 0.65). Long-term studies of EMERGENT-4 and EMERGENT-5, covering 52 weeks of therapy, showed durability of the therapeutic effect, as well as a stable tolerability profile with no cumulative toxicity (Psychiatrictimes, 2025). Adverse effects - mainly on the gastrointestinal side - were mostly mild to moderate and did not lead to a significant rate of therapy discontinuation, in contrast to previous therapeutic options (Kaul et al., 2024a; Kaul et al., 2024b; Brannan et al., 2021; Correll et al., 2022; Lieberman et al., 2005).

The absence of the usual extrapyramidal symptoms, hyperprolactinemia or metabolic syndrome typical of D_2_ antagonists is of high importance. This makes KarXT an encouraging alternative for patients who are poorly tolerant of existing antipsychotics, as well as those with drug-resistant schizophrenia, for whom clozapine currently remains the only treatment option (Fenton and Kang, 2023).

Of particular interest in the context of further development of cholinergic therapy is TerXT, a new prodrug form of xanomeline and trospium being developed by Terran Biosciences. KarXT requires twice-daily administration, which is a significant therapeutic problem in patients with schizophrenia. TerXT is designed as a drug taken once a day in oral form and in a depot version (TerXT LAI) - administered intramuscularly with prolonged action. Both formulations are expected to provide more stable concentrations of the drug, reduce side effects and improve treatment adherence (Beaney, 2024).

From a systemic perspective the cost of therapy can be a significant challenge. According to estimates, the annual price of KarXT in the U.S. is about $22,500, which exceeds ICER’s recommended cost-effectiveness threshold ($16,000-20,000 per QALY). Nevertheless, the drug has been deemed “promising but inconclusive” (Icer, 2025). The manufacturer, Karuna Therapeutics (now part of BMS), is considering patient support programs and price negotiations, which may facilitate the drug’s availability on the market. Importantly, Bristol speculates that 80% of the population treated with KarXT will be covered by Medicare and Medicaid within 12–18 months of the drug’s launch (Satija et al., 2024).

Although no direct head-to-head trials comparing KarXT with typical or atypical antipsychotics are currently available, several clinically relevant distinctions can be drawn based on mechanism of action and observed safety profiles. Unlike typical antipsychotics, which exert antipsychotic effects primarily through D_2_ receptor antagonism and are associated with a high risk of extrapyramidal symptoms, KarXT does not directly interfere with dopaminergic transmission, thereby largely avoiding motor side effects and hyperprolactinemia (Yohn et al., 2022; Paul et al., 2024; Kaul et al., 2024a; Kaul et al., 2024b; Brannan et al., 2021; Leucht et al., 2013; Haddad and Wieck, 2004).

Compared with atypical antipsychotics, KarXT similarly demonstrates efficacy in reducing positive symptoms, but with a distinct adverse event profile characterized predominantly by transient gastrointestinal effects rather than metabolic disturbances, weight gain, or sedation. Importantly, muscarinic M_1_ receptor engagement offers a plausible advantage in addressing cognitive and negative symptoms, domains in which both typical and many atypical antipsychotics show limited efficacy. These differences suggest that KarXT may occupy a complementary therapeutic niche rather than directly competing with existing dopamine-based agents (JMCP, 2026; Lobo et al., 2022; Icer, 2025).

The prespecified secondary measures were also negative; however, *post hoc* analyses indicated a greater effect when background therapy excluded risperidone. The add-on result suggests that efficacy demonstrated in acute monotherapy settings may not translate into short-term adjunctive benefit for residual symptoms (Weiden et al., 2022). These findings highlight important limitations of the current evidence and underscore the need for further well-designed studies, particularly in patients with predominant negative symptoms or cognitive impairment, and with longer treatment durations.

Overall, KarXT represents a potentially transformative therapeutic option for schizophrenia with a unique mechanism of action that may provide significant benefits in addressing negative symptoms and cognitive deficits. It offers a favorable safety profile, a real improvement in patients’ functioning and a potential change in the approach to schizophrenia pharmacotherapy in the coming years. Its further development, monitoring of efficacy in real-world populations, and regulatory decisions regarding availability and price will determine its place in future treatment standards.

## Glossary

AEs: adverse effects
ARISE: Phase 3 adjunctive trial evaluating Cobenfy (xanomeline–trospium) in adults with schizophrenia inadequately controlled on atypical antipsychotics
BMS: Bristol Myers Squibb
cAMP: cyclic adenosine monophosphate
CB2: cannabinoid receptor type 2
CGI-S: Clinical Global Impression–Severity
**C**_**max**_: maximum plasma concentration
CNS: central nervous system
DSM-5: *Diagnostic and Statistical Manual of Mental Disorders*, 5th Edition
DSM-IV: *Diagnostic and Statistical Manual of Mental Disorders*, 4th Edition
EMERGENT: program of clinical trials evaluating KarXT in schizophrenia
EMERGENT-1, EMERGENT-2, EMERGENT-3, EMERGENT-4, EMERGENT-5: individual trials within the EMERGENT program
EPS: extrapyramidal symptoms
FDA: Food and Drug Administration
GABA: gamma-aminobutyric acid
**G**_**i/o**_: coupled receptors inhibit adenylyl cyclase
**G**_**q**_: coupled receptors activate PLC
ICD-10: *International Classification of Diseases*, 10th Revision
ICER: Institute for Clinical and Economic Review
KarXT: xanomeline-trospium
KCNQ 2/3: subunits 2 and 3 of potassium voltage-gated channel
LAI: long-acting injectable
LSMD: least squares mean difference
**M**_**1**_: muscarinic acetylcholine receptor subtype 1
**M**_**4**_: muscarinic acetylcholine receptor subtype 4
**M**_**5**_: muscarinic acetylcholine receptor subtype 5
NDA: New Drug Application
OAB: overactive bladder
PANSS: Positive and Negative Syndrome Scale
**PIP**_**2**_: phosphatidylinositol 4,5-bisphosphate
PLC: phospholipase C
RCT: randomised controlled trial
QALY: quality-adjusted life year
QT: QT interval
TEAEs: treatment-emergent adverse events
TerXT: Terran Biosciences xanomeline-trospium
**T**_**max**_: time to maximum plasma concentration
T½: elimination half-life
VTA: ventral tegmental area
WHO: World Health Organization
